# Statistical Study on Human Temperature Measurement by Infrared Thermography †

**DOI:** 10.3390/s22218395

**Published:** 2022-11-01

**Authors:** Michal Švantner, Vladislav Lang, Jiří Skála, Tomáš Kohlschütter, Milan Honner, Lukáš Muzika, Eliška Kosová

**Affiliations:** New Technologies-Research Centre, University of West Bohemia, 301 00 Pilsen, Czech Republic

**Keywords:** infrared thermography, human temperature, body temperature, thermographic measurement, infectious diseases, thermal imaging, infection control, emerging diseases, medical thermography, fever screening

## Abstract

Increased temperature in humans is the symptom of many infectious diseases and it is thus an important diagnostic tool. Infrared temperature measurement methods have been developed and applied over long periods due to their advantage of non-contact and fast measurements. This study deals with a statistical evaluation of the possibilities and limitations of infrared/thermographic human temperature measurement. A short review of the use of infrared temperature measurement in medical applications is provided. Experiments and statistics-based evaluation to confirm the expected accuracy and limits of thermography-based human temperature measurement are introduced. The results presented in this study show that the standard deviation of the thermographic measurement of the eyes maximum temperature was 0.4–0.9 °C and the mean values differences from the armpit measurement were up to 0.5 °C, based on the used IR camera, even though near ideal measurement conditions and permanent blackbody correction were used. It was also shown that a certain number of outliers must be assumed in such measurements. Extended analyses including simulations of true negative/false positive, sensitivity/specificity and receiver operating characteristics (ROC) curves are presented. The statistical evaluation as well as the extended analyses show that maximum eyes temperature is more relevant than a forehead temperature examination.

## 1. Introduction

Increased temperature or fever in humans can be an indication of many infectious diseases and temperature diagnostics is thus an important and widely used method to provide an indication of these diseases [1]. A body core temperature, which refers to the body’s internal organs, is the decisive quantity. It is influenced by various effects (e.g., age, sex, health conditions, etc.), however, the usually reported range is 36–36.9 °C (there are some differences based on a source, e.g., [2,3,4]). An increased temperature is considered 37–38 °C and a fever is more than 38 °C. Peripheral body temperature (i.e., skin level), can vary significantly depending on location, health, local health conditions, or ambient conditions.

The reference methods for human core temperature measurement are contact-based methods [5], which use thermometers or infrared-based probes. These methods can be invasive or non-invasive. Rectal, esophagus, pulmonary artery or urinary bladder diagnostics belong to invasive methods, from which the rectal measurement is considered the most practical and accurate to determine the body core temperature [5]. The invasive medical procedures are the most reflective, but they can be mostly applied in medical facilities only. Temperature measurement in mouth (oral), ear (tympanic) or armpit (axilla) belongs to the non-invasive methods. They are easier to use and can thus also be at home, but they are considered less accurate. Procedures of use, equipment requirements, and interpretation of results for these methods are described e.g., in publications by Moran and Mendal [5], Werner [6] or in the standard EN ISO 9886 [7].

The contact-based methods are mostly not practical for screening in public spaces. Thus, infrared (IR) non-contact methods have become popular and have been developed and widely applied for human temperature screening and diagnostics. Results of the IR screening of humans have however not been fully satisfying and the effectiveness of the IR methods for these purposes has been sometimes disputable. This study presents experimental research focused on statistics-based confirmation of possibilities and limits of thermography-based screening, which is often used in public spaces during infectious pandemics. The paper follows and extends the conference proceedings [8] published within the IEEE International Workshop on Metrology for Living Environment—MetroLivEnv 2022.

## 2. Overview and State of the Art

Thermography have been used in medical applications for many years [1,9], for example, for diagnostics of inflammation, diabetic diseases or in dentistry. Infrared thermography (IRT) methods are non-contact, fast and very effective if qualitative thermography is sufficient, i.e., temperature contrast differences are to be determined. However, human body temperature measurement requires a high absolute accuracy of about ±0.1 °C [5,10], which is significantly higher than a standard accuracy of ±2 °C of the most common micro-bolometer-based infrared cameras. Another disadvantage of the thermographic methods is that only skin is measured [11], but the core temperature should be evaluated. Many studies have thus been focused on the reliability and accuracy of the thermographic diagnosis of human body temperature.

A reliability study for handheld infrared thermometers, which have been widely used in homes, hospitals and, for example, also in public spaces during the COVID-19 pandemic, was introduced in [12]. Another publication presented research dealing with a suitable alarm temperature and measurement distance for mass screening [13], the influence of the angle and distance on the face temperature measurement [14] or with the classification of factors influencing human body temperature measurement by IRT methods [15]. The standard [10] defines measurement procedures and requirements as, for example, devices accuracy, environment, measurement configuration, blackbody use, etc. It defines, among other things, that a measured person should be thermally stabilized, the face should not be covered, the temperature should be read in the inner canthus (a corner of the eye where the upper and lower eyelids meet) and a reference blackbody with a sufficient accuracy should be always used because of the limited accuracy of the infrared cameras. However, compliance with these conditions leads to a near laboratory measurement and it is hardly possible to comply with these conditions in practical use for mass screening.

Despite these drawbacks, a big advantage of the IRT methods is that they can be easily used at a safe distance in public spaces. The methods for screening purposes during infectious epidemics have been thus developed and widely applied. Their development accelerated particularly during the SARS epidemic in 2002 [16,17] and subsequently during the H1N1 epidemic in 2009. For example, a study about the efficiency of the infrared screening of travelers during epidemics was published by Bitar et al. [18] in 2009, research confirming that thermography can be usable for the mass screening of fever was published by Nguyen et al. [19] in 2012 and a study dealing with a use of the IRT for mass screening at Narita airport (Japan) during the H1N1 epidemic was published by Nishiura and Kamiya [20] in 2011. Interest in infrared methods for human body temperature screening grew with subsequent epidemics of MERS-CoV in 2012 and Ebola in 2014. This growth was connected with an increase in world population mobility, discussions about a possible global pandemic and, thus, the need for a useful tool for mass temperature screening in airports and other checkpoints [21,22]. Finally, infrared methods were also widely used during the COVID-19 pandemic, which started in 2020 [23].

Approaches based on IRT monitoring of persons in large public spaces [24] are quite inaccurate. Another approach uses a “floating reference”, to which the maximum face or inner canthi temperature is compared. The floating reference is derived as an average of measured persons in a certain time-period that makes it possible to suppress some environmental condition changes. The effectiveness of these methods can however decrease due to some specific factors, for example, due to face masks or glasses. A statistical rating of these approaches is more important than an evaluation of the accuracy of the used measurement systems, which is determined in terms of a laboratory-based experiment. The statistical rating is mostly focused on sensitivity and specificity evaluation (relations between true positive, true negative, false positive and false negative) in terms of identification of persons with higher temperatures or fever. Verification of the reliability of these methods requires simultaneous diagnosis by a certain reference method and also a certain number of “positives”, that is, some measured persons with a higher body temperature or fever. It makes the organization of such experiments very complicated. Results of the published studies related to mass screening were however sometimes disputable, and some authors were doubtful about an efficiency of the screening (in general) at borders to prevent an introduction of diseases into a country [25]. The real benefits of mass implementation are therefore still unsatisfactory, and research on measurement methods and evaluation procedures continues.

It is worth noting that even if most of the above-mentioned infectious diseases can manifest themselves in a higher body temperature, this is not the case in all patients and not the case in all phases of diseases. This makes the IRT-only examination even less effective. This was confirmed in a study [26], which is related to screening methods of influenza patients. The research summarized that a positive predictive value of IRT screening is in the wide range of 3.5–65.4% according to different studies. The study [26] showed that a very high detection effectiveness up to 93% of positive predictive values can be achieved by combination of different diagnostics methods (for example heart and breath rate). Authors of this study also introduced the implementation of a screening radar system combining different diagnostics methods and neuron networks/fuzzy grouping evaluation [27,28]. Such research is however not the subject of this work.

This study follows on from previously published conference proceedings [29] and it is based on and significantly extends the conference proceedings [8]. It is focused on an experimental statistical determination of the possibilities and limits of human body temperature measurement by thermographic methods. Several research studies about this topic have already been published. For example, research about IRT effectiveness in the detection of patients with a fever was presented in [13,20] or in the review paper [18]. The clinical accuracy and calibration of human IRT measurement was studied in [30,31] and research about requirements for IRT devices for medical applications was presented in [9] or in review paper [1]. Specific requirement and guides for human IRT measurement was defined in the standard [10] or in the most pertinent factors review [32]. Laboratory studies on the different effects on human IRT temperature measurement have been also already published (for example, the study related to angles and distance of the measurement [14] or the review study about different factors influencing the use of infrared thermography in humans [15]). In contrast to these studies, this work is focused on statistical evaluation of human IRT measurements performed at different conditions. The experiments were carried out over several months and the goal was not to analyze individual persons and/or measurements, but to statistically evaluate and confirm mean values, variability and reliability of IRT human body temperature measurements on a healthy group of testers. The variance in this case included devices uncertainty as well as differences between individual testers and differences of the testers during a measurement period. This approach could be more relevant for real applications than a precisely guided laboratory experiment. Extended analyses including simulations of true negative/false positive, sensitivity/specificity and receiver operating Characteristics (ROC) [33] curves are also provided in addition to standard statistical evaluations.

## 3. Experimental Procedure

Experiments presented in this contribution were focused on establishing the possibilities and limits of IRT human body temperature measurement in near laboratory conditions. Tests were performed simultaneously in two buildings of the University of West Bohemia and the persons participating in the testing (testers) were volunteers, who consisted mostly of staff members and students in the buildings. The testers could perform the measurement repeatedly. The goal was to evaluate the measurement procedure and equipment; thus, the condition or health state of the individual testers was not monitored. All the testers were healthy, and a group of testers was a mix of men and women. The measurement was strictly anonymous and the ratio of men and women or the frequency of measurement of the individual testers were not registered (tens of different testers participated in the measurement).

The experiments started in November 2020 and continued to April 2021. The instructed testers carried out a self-measurement repeatedly during the experiment period. They measured their temperatures simultaneously by an infrared camera (their face) and by an armpit thermometer. Requirements defined by the standard were not strictly followed, however, all testers were thermally stabilized inside the building and instructed on how to carry out the IRT measurement in the right way. They were instructed, for example, to take off glasses or face masks, to carry out measurements after at least 30 min of being in the building, not to wash their face or to drink/eat before the measurement, etc. Nevertheless, specific conditions in individual locations of the building (including a temperature in a measurement room) and conditions or activities of the testers before the measurement were neither specified nor monitored.

**Figure 1:**
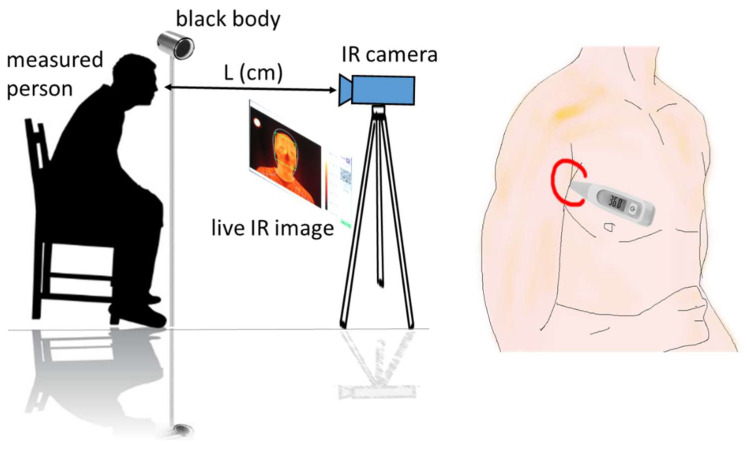
Scheme of the human temperature measurement by infrared thermography and by an armpit thermometer.

A scheme of the experiment is shown in Figure 1. The measurement locations were in a room at standard-office non-controlled conditions (temperature 20–30 °C, humidity 20–50%). The measurements were performed in such a way that an IR camera and a reference blackbody (BB) were at fixed positions. The BB was always in the field of view of the camera and took about 2% of a recorded scene. The testers took a specified position near the BB in the field of view of the IR camera so that their faces occupied about 30% of the scene. A non-reflective wall or fabric surface was in the background of the scene. A tester saw a monitor with a live IR view, which was located near the IR camera and helped to correct their position. A thermographic image was recorded and saved automatically after a control software recognized the tester in the specified position. The tester subsequently measured and registered their body temperature by the armpit thermometer (APT). The distance (L) of the IR camera from the tester depended on the camera and lens used (this will be specified next).

The experiments were performed using FLIR A315, FLIR A615 and LabIR IR cam-eras, which are based on FLIR Lepton module. The IR cameras were non-cooled micro-bolometers based devices working in a wavelength range 7.5–13 μm. The FLIR A315 IR camera had the resolution 320 × 240 pixels, thermal sensitivity/NETD < 0.05 °C at +30 °C, accuracy ±2 °C or ±2% of reading. A standard lens FOV 25° × 18.8° with the focal length 18 mm was used and the distance of the tester from the IR camera was about 100 cm. The FLIR A615 IR camera had a resolution of 640 × 480 pixels, thermal sensitivity/NETD < 0.05 °C at +30 °C, accuracy ±2 °C or ±2% of reading. A telescopic lens FOV 15° × 11° with the focal length 41.3 mm was used and the distance of the tester from the IR camera was about 230 cm. The LabIR device is a FLIR Lepton 3.5 detector-based IR camera. It has a resolution of 160 × 120 px, NETD < 0.05 °C, accuracy ±5 °C and FOV 57° × 71°. It was about 60 cm from the tester. A reference blackbody Kleiber KBB 40 with a reference temperature of 40 °C and emissivity of 0.98 ± 0.004 was used for each measurement. It had the aperture of 50.8 mm, uncertainty of 0.4 °C, repeatability of 0.2 °C and stability of 0.1 °C. The measurements were made with the emissivity 0.98 [31] set on the IR camera. The distance (L) of the IR camera from the tester depended on the camera and lens used (this will be specified next).

The testers measured and registered their body temperature with the armpit thermometer (APT) immediately after the IR measurement. The electronic thermometers Microlife MT850 and Hartman Thermoval Standard (a ±0.1 °C accuracy for both) were used for the armpit body temperature measurement. The testers were instructed to use the APT according to the user guide of the devices and the following rules:

- Clean/disinfect the thermometer and turn it on.
- Insert the probe tip (which is partly flexible for these models) into the center of the armpit to be in a full contact with the skin.
- Put down the arm against the body while applying slight pressure to fix the thermometer.
- It is necessary to be careful so there is no clothing/fabric between the tip of the thermometer and the skin.
- Hold the thermometer for about 2 min (the sound of the thermometer should not be taken into account).
- Remove the thermometer and read/register the measured value.
- Clean/disinfect the thermometer again.

Collected data of both IRT and APT measurements were evaluated manually.

## 4. Results

In total, about 750 valid records were acquired during the testing period. The goal of this study was not to find differences between the various ages or genders, etc. Thus, these pieces of information were not collected. However, we estimate that the age of the testers was in a wide range between 18 and 65, with a higher number of testers between 18 and 50. A higher number of men participated in the testing at the 1st measurement location (FLIR A315 and LabIR cameras), where about 15–20 testers performed the tests repeatedly. A higher number of women is estimated at the 2nd measurement location (FLIR A615), where we estimated participation of about 50–100 testers, who performed the tests with fewer repetitions. Thus, we also estimate that there was roughly a similar number of men and women in the data set.

Several values were evaluated for each infrared image (thermogram): maximum and minimum face temperature, location of the maximum, maximum temperature at inner cantus region, forehead temperature, BB temperature, and background temperature were evaluated and analyzed together with the connected armpit body temperature of the tester (the selected results only are presented in this contribution). The evaluation is not made in terms of individual values comparison, but statistical values are evaluated.

**Figure 2:**
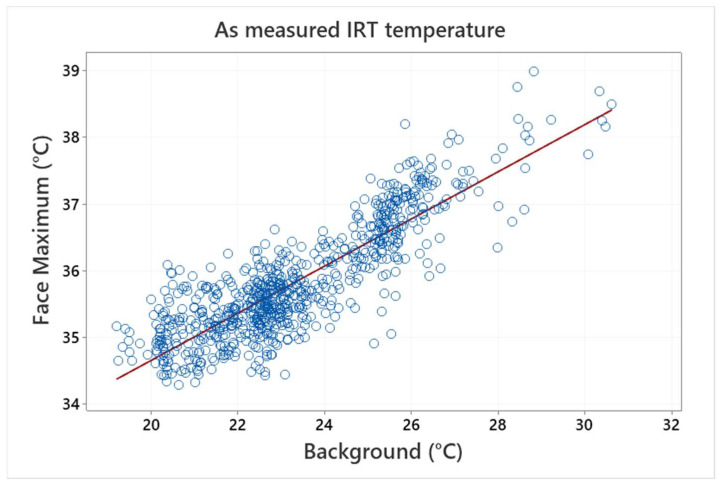
Dependence between IRT measured face temperature and background temperature without corrections—as measured values (symbols) and a linear regression (line) are shown.

Micro-bolometer-based thermographic devices are often influenced by ambient temperature. This was also observed in this study, as a clear dependence between the IRT measured face temperature and the background temperature was found. The measured background temperature was 23.06 ± 0.99 °C (minimum/maximum~20.51/27.73 °C). The relationship between the background and maximum face temperatures is illustrated (as measured—without any correction) in Figure 2.

As reported in [29], this effect can be suppressed by a blackbody correction. It decreases the significance of the correlation (influence of ambient conditions), decreases the standard deviation of mean values (in [29] it was 0.43 °C and 0.36 °C before and after the correction for FLIR A315 IR camera, respectively), and brings the measured maximum face temperature mean value closer to the armpit measurement.

**Figure 3:**
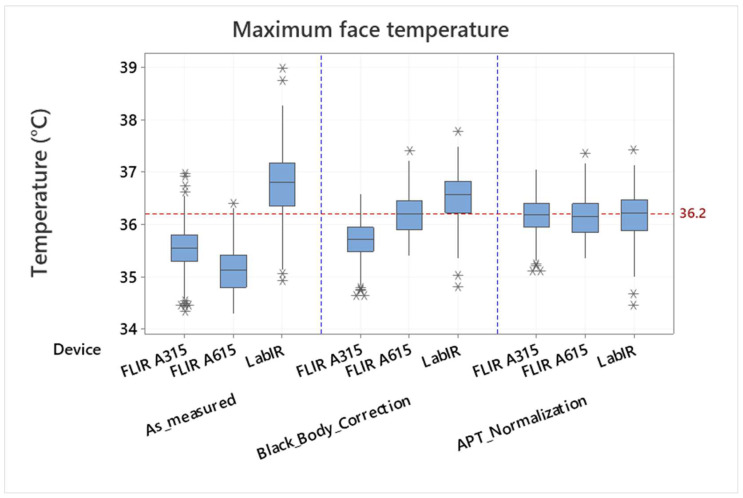
Comparison of face maximum temperature measured by the FLIR A315, FLIR A615 and LabIR IR cameras: as measured (As_measured), corrected by the blackbody (Black_Body_Correction) and normalized according to the armpit thermometer mean values measurement (APT_Normalization). Outliers are identified by asterisks (*).

The measured blackbody temperature was 39.72 ± 0.89 °C (minimum/maximum ~ 38.14/41.71 °C, the 95% confidence interval for mean was 39.68–39.77 °C, the expected blackbody temperature was 40 °C). The blackbody correction effect is demonstrated in Figure 3, where a maximum face temperature measured by the FLIR A315, FLIR A615 and Lepton IR cameras is shown: as measured and after the blackbody correction (see “As_measured” and “Black_Body_Correction”, respectively). It is however evident from the boxplots presented in Figure 3, that even though the results are better if a blackbody correction is used, there are still differences between the individual IR cameras, and the IRT measurement still does not correspond to the measured armpit temperature (the mean value of 36.16 °C is shown by the reference line). It means that the measured values are dependent on the individual devices and their measurement configurations (e.g., the distance from the measured object). Assuming that the measurement configuration does not change during the experiment it is possible to make a normalization according to a mean value of the armpit thermometer measurement and to mean values of the individual devices’ IRT measurement. The normalized values are shown in Figure 3, part “APT_Normalization”. Standard deviations and variance of the values are different for the individual devices (measurement configurations), however, their mean values correspond to each other and can be thus compared or processed together.

**Figure 4:**
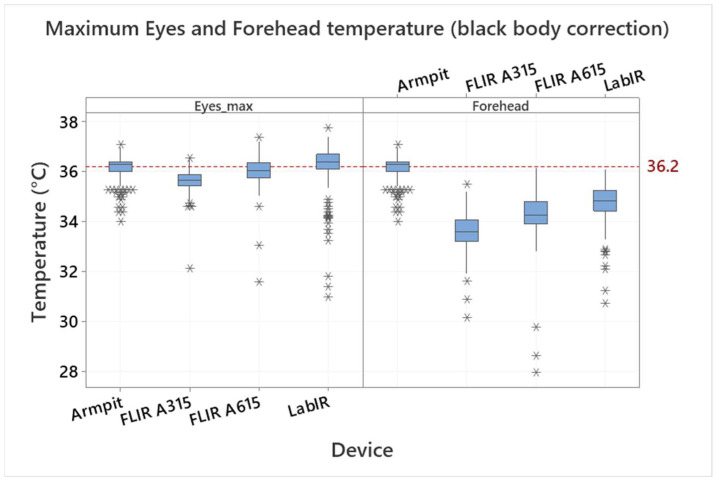
Comparison of eyes maximum (inner canthus) and forehead temperature measured by the FLIR A315, FLIR A615 and LabIR cameras (blackbody correction applied) with the body temperature measured by the armpit thermometer. Outliers are identified by asterisks (*).

**Figure 5:**
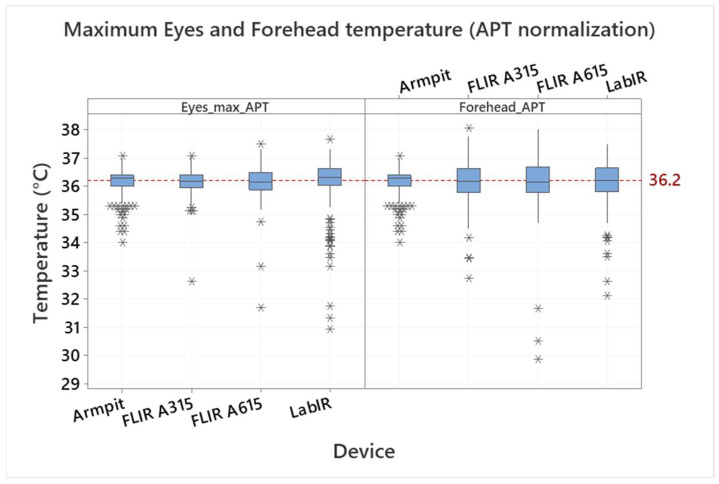
Comparison of eyes maximum (inner canthus) and forehead temperature measured by the FLIR A315, FLIR A615 and LabIR cameras (blackbody correction and armpit temperature normalization applied) with the body temperature measured by the armpit thermometer. Outliers are identified by asterisks (*).

The effect of the normalization is demonstrated in Figure 4 and Figure 5, where the eyes maximum temperature and forehead temperature are compared for individual devices. Figure 4 shows the eyes maximum temperature and the forehead temperature measured by the IRT (by devices) after the blackbody correction (normalization not applied); the IRT measurement is also compared with the armpit temperature measurement. The eyes area maximum temperatures are 35.65 ± 0.41, 36.05 ± 0.58 and 36.23 ± 0.90 °C by the FLIR A315, FLIR A615 and LabIR IR cameras, respectively. There are even bigger differences in the case of the forehead temperatures, which are 33.59 ± 0.68, 34.28 ± 0.91 and 34.77 ± 0.76 °C for the FLIR A315, FLIR A615 and LabIR IR cameras, respectively. It is also evident, that the forehead IRT temperature measurement does not correspond to the real core body temperature, represented in our case by the armpit/axillary measurement. There is an offset, which is from 1.39 to 2.57 °C, based on the IR camera. It is worth noting that this also applies for any handheld IR medical thermometers applied to forehead, as it was presented, e.g., in [34]. As shown in Figure 5, the mean values after the normalization of all the IRT measurements are the same and the only differences are in the standard deviations and variations, which correspond to the date before the normalization.

**Figure 6:**
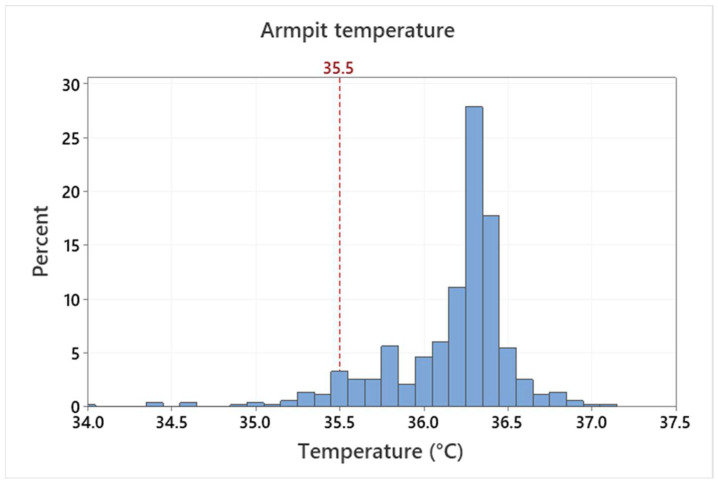
Histogram distribution of the armpit temperature measurement.

**Figure 7:**
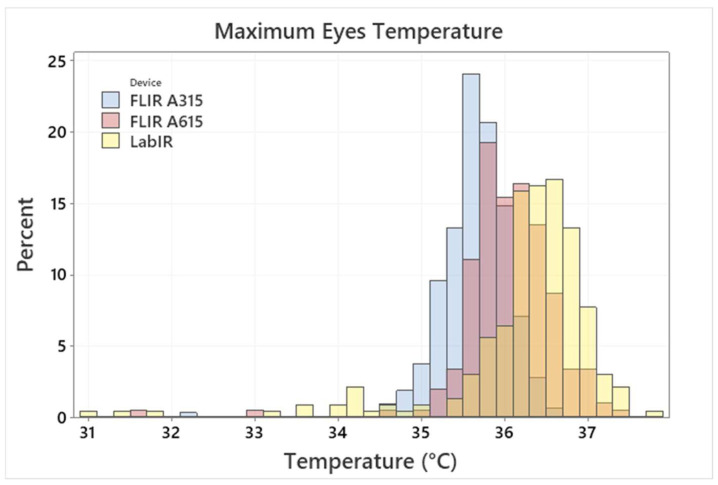
Maximum eyes temperature by the FLIR A315/615 and LabIR IR cameras histogram distributions. (The diagram represents combination of histograms of 3 devices, 3 colors in the legend indicate 3 individual devices. Other colors in the graph represent overlaps.)

The mean armpit temperature of all measurements is 36.16 ± 0.38 °C. Some scatter, outliers and distribution skewness can be registered, as shown in Figure 6. This variance is caused by natural differences (between testers and in course of time of individual testers) as well as by a non-perfect armpit temperature measurement due to a self-measurement procedure. The testers measured themselves based on the defined rules without the assistance of a medical personnel. The mean value is in accordance with other published results, however, some outliers in the data are unrealistically low. Based on measurement conditions, this points to non-correct measurement procedures rather than the real temperature of the testers. The normal temperature range reported, e.g., in [2] is from 35.5 to 37.0 °C. It is therefore questionable, if values under 35.5 °C should be assumed as valid. Such assumption would lead to the mean value of 36.22 ± 0.29 °C. However, the differences between the two values are quite small and the variation of the armpit temperature measurement is significantly smaller compared to face IRT measurement. The distribution for maximum eyes temperature is shown in Figure 7.

Differences between the individual IR cameras are caused by their technical characteristics, for example, resolution, their response to ambient changes, etc., (an analysis on the factors influencing the IRT measurement was presented, e.g., in [35]). The discrepancy between APT and IRT measurement is caused by above-mentioned reasons and, additionally, by physiological reasons and some differences of conditions in the measurement room, ambient conditions in the location of the testers or of testers activity before the measurement. It is significant especially in the case of the forehead IRT measurement.

The eyes region maximum temperature, which should be related to the inner canthi, is usually assumed the most relevant IRT measurement location and the maximum face temperature position. However, analysis of the results in Table 1 shows that 74.4% of maximum face temperature values were related to the eyes region, 6.7% to the neck region, 9.6% to the mouth region, 2.8% to the forehead and temple region and 6.6% to other face locations. This can be caused by external factors or by minor injuries to or inflammation of the face. However, there are not significant differences between the inner canthi and other positions, at which a maximum face temperature was detected.

**Figure 8:**
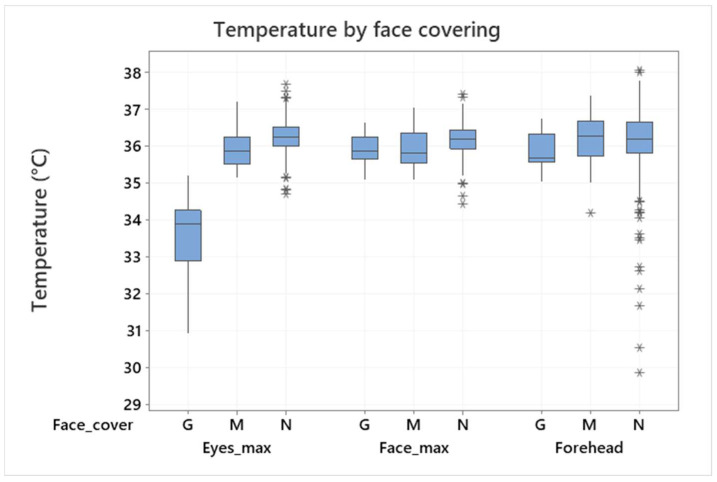
Maximum eyes/face temperature and forehead temperature in relation to face covering: G–glasses, M–facemask, N–none (armpit normalization applied). Outliers are identified by asterisks (*).

Thus, the eyes and face maximum temperatures can be assumed as relevant if there is not another reason to set an exact measurement location. This statement is however valid only in the cases where the eyes are not covered, e.g., by glasses. In this case, the maximum face temperature can be hidden, and the temperature examination can be incorrect. This is demonstrated in Figure 8, which shows differences of the mean values if the face is partially covered by glasses. (It was discovered during the evaluation that a small number of testers kept their glasses on although they were instructed to perform the measurement without them.) The conclusion of this analysis is that both maximum face temperature and maximum eyes temperature are relevant and can be assumed for an examination, but eyes shall not be covered (by glasses) in any case. In contrast, this study did not confirm the importance of a face mask or a small defocusing of the IR camera (mostly due to movement or the incorrect positioning of the testers).

The results presented in this section were achieved by the experiments carried out under some regulations and limitations, however, some typical sources of variation (e.g., between testers, for one tester in a course of time, etc.) and uncertainty (e.g., self-measurement, different cameras and measurement configurations, the condition of testers before and during the measurement, etc.) were included. It was not the goal of this study to evaluate influence of the individual uncertainty sources. It should also be understood, that the results may vary under other testing conditions, e.g., different environments or different group of testers (age, gender, condition, etc.). However, the importance of the blackbody correction, possible influence of the environment (background) or influence of device properties and measurement configuration were demonstrated clearly and quantified for this particular study. The presented statistical results (variations, standard deviations, and outliers) can be considered as typical for similar experimental configurations. They can also be considered as limiting, because the conditions of most of the publicly applied infrared screenings are worse than the conditions defined for this research.

## 5. Extended Evaluation

**Figure 9:**
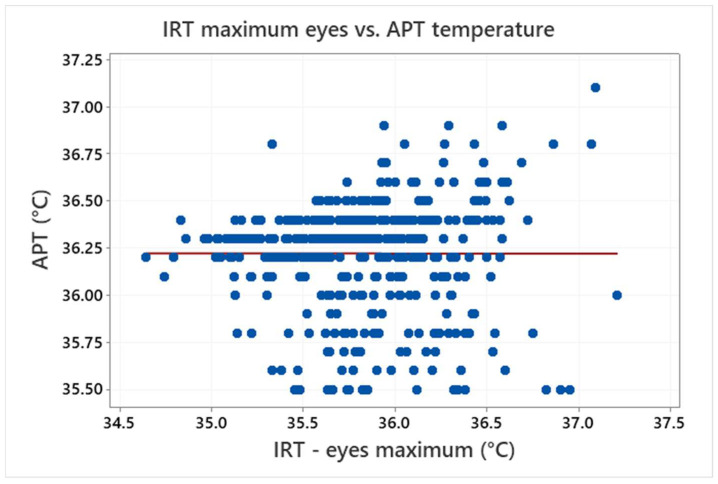
Relationship between the IRT eyes maximum temperature and APT temperature (the symbols represent measured values and the line shows their linear regression).

This study does not examine the state of health of the testers and it is also assumed that the testers were healthy persons only. This leads to a very small range in the temperature of the testers (APT), which is in a scale of the scatter of the measured data. As a result, Figure 9 shows that it is not possible to find a relationship between the IRT maximum eyes temperature and APT temperature (the data with APT values under 35.5 °C were assumed as invalid and thus excluded from the analyses for the purposes of the extended evaluations).

**Figure 10:**
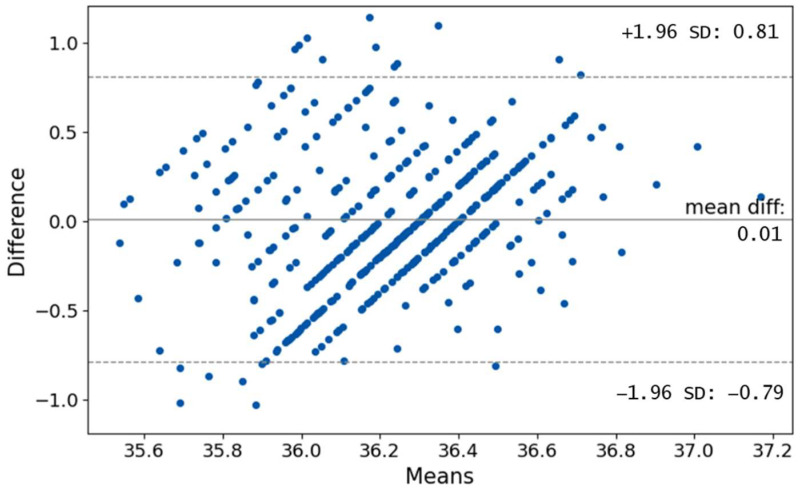
Relationship between the IRT eyes maximum temperature and APT temperature.

A Bland–Altman plot which is often used to visualize the differences in measurements between two different instruments is shown in Figure 10. We can see that most of the differences are in limits of ±1.96 SD, which translates to +0.81 °C and −0.79 °C. Plenty of points are outside the region. Normally, the difference of ±1.96 SD is not clinically important, but in the case of human core temperature, this difference may be crucial as it can greatly influence whether the person is considered ill. It can be seen that the bias is 0.01. Such small values however can be expected due to the fact that the data of the IRT measurements were normalized (see Figure 4 and Figure 5). Furthermore, we can notice obvious lines under angle 45°. This is caused by the lower decimal resolution of the APT (one decimal resolution).

This study cannot predict what relationship might be found if a greater range of temperatures from the testers were available (including, e.g., hypothermic patients and patients with a fever). A study including patients with a body temperature higher than 39.5 °C was presented in [16], where a stronger relationship between the IRT (eyes range) and body (ear clinical thermometer) was found. Such a study however has to be performed under very specific conditions in a medical facility. This is not a goal of our study, which is focused on conditions that are more natural. In any case, the results published in [16] also show a scatter, which leads to R2 values of 0.55 for eyes region and 0.50 for forehead region. This indicates that a scatter of the IRT measurement is limiting for a precise body temperature examination.

**Figure 11:**
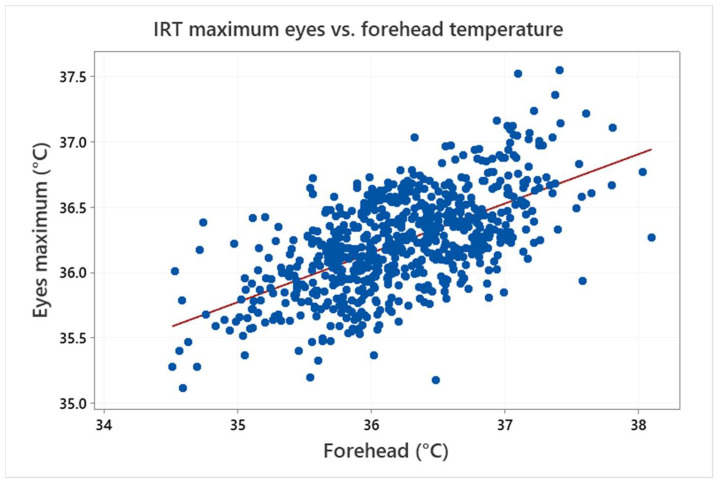
Relationship between the forehead and maximum eyes temperature measured by IRT (the symbols represent measured values and the line shows their linear regression).

This is also demonstrated in Figure 11, which shows a clear relationship between the IRT forehead and maximum eyes temperature. The linear regression analysis confirmed that the association is statistically significant (*p*-value < 0.05), but the R2 value is about 0.37 only, which means that the model does not fit the data well enough. In conclusion, the results confirm a strong relationship between these two values, but they show that, due to the data scatter, the forehead temperature measurement can be hardly used for an accurate determination of the maximum eyes temperature or the body (armpit) temperature of the examined testers.

The main reason for using thermography for human temperature measurement/screening is to find people with an increased temperature or a fever (by a fast, non-invasive and non-contact way). The “true” body temperature is in our case introduced by the APT measurement. However, based on the results of this study, the IRT data cannot be reliably connected with the APT by a simple relation. Thus, a statistical description of the ability and reliability of the IRT to indicate “positive” cases in relation to the true body temperature and a selected threshold is required. For these reasons, determination of sensitivity, specificity and ROC is often used in similar cases [16,20].

**Figure 12:**
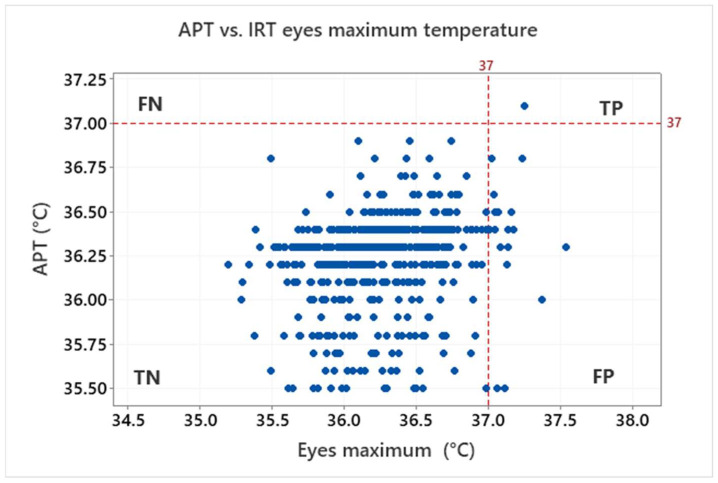
Graphical illustration of true positive (TP), true negative (TN), false positive (FP) and false negative (FN) for the IRT eyes maximum temperature in relation to the APT with the threshold 37 °C.

**Figure 13:**
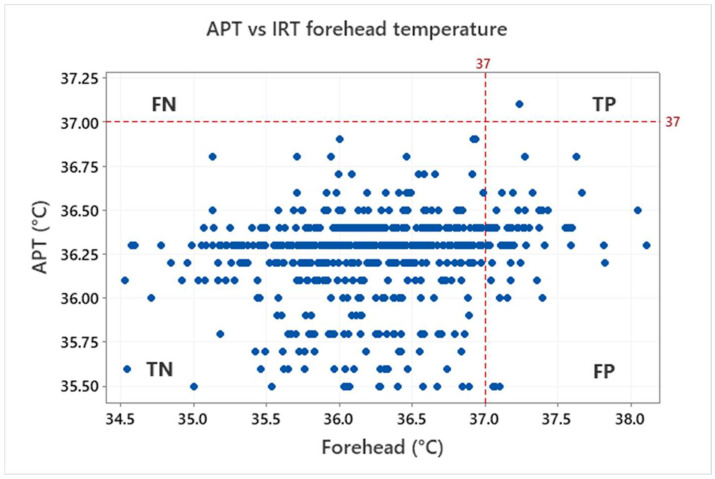
Graphical illustration of true positive (TP), true negative (TN), false positive (FP) and false negative (FN) for the IRT forehead maximum temperature in relation to APT with the threshold 37 °C.

The above-mentioned characteristics are derived based on the ratio of true positive (TP, both IRT and APT over the threshold), true negative (TN, both IRT and APT under the threshold), false positive (FP, IRT over and APT under the threshold) and false negative (FN, IRT under and APT over the threshold). Results for the threshold 37 °C, which is often used to identify sick persons, are shown in Figure 12 for the eyes maximum and Figure 13 for the forehead IRT measurement.

Based on the assumption of healthy testers (i.e., the testers should not have a body temperature higher than 37 °C), no FN was indicated. One tester had a body temperature (APT) higher than the 37 °C threshold. Although this was also identified by both the forehead/maximum eyes IRT (i.e., TP), it is statistically irrelevant (0.2%). Most of the testers are in the TN group: 97.0% for eyes maximum, 88.7% for forehead. However, some of the testers are also in the FP group: 2.8% for eyes maximum, 11.2% for forehead. That means the ratio of false positives is appreciable and cannot be neglected especially for the forehead IRT measurement.

**Figure 14:**
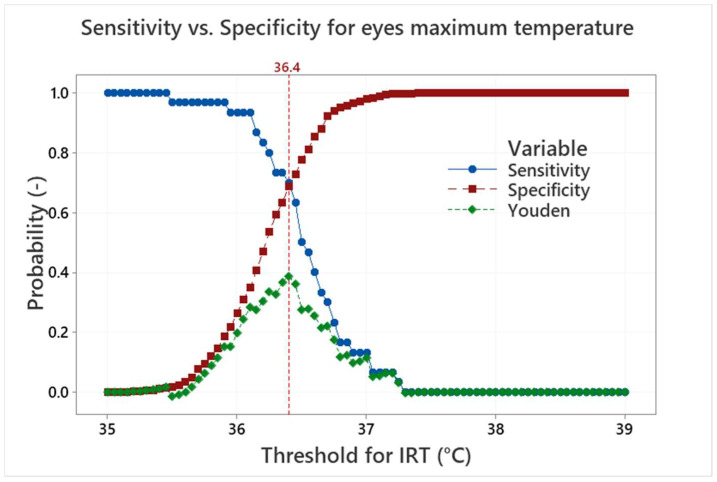
Sensitivity, specificity and Youden index curves for the maximum eyes temperature and the APT threshold of 36.5 °C.

**Figure 15:**
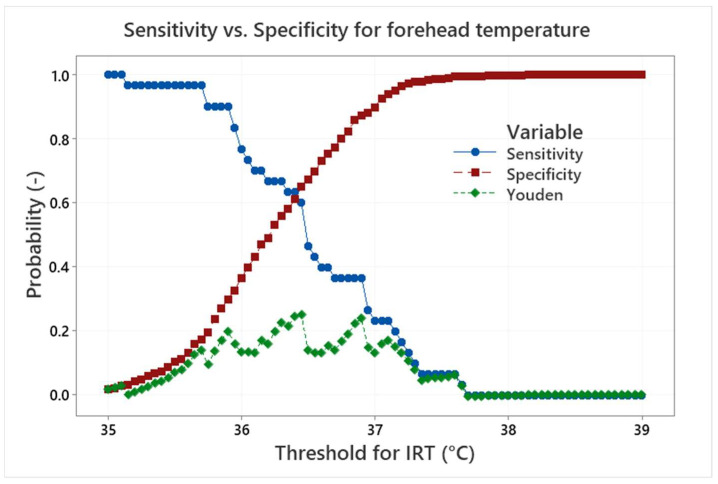
Sensitivity, specificity and Youden index curves for the forehead temperature and the APT threshold of 36.5 °C.

Sensitivity and specificity can be represented as probability curves (S-S curves) showing how different IRT thresholds are effective to find testers with an APT above a given real body temperature threshold. Sensitivity is a measure of how well the IRT measurement can identify true positives (correctly detect persons with real body temperature higher than the threshold). Specificity is a measure of how well the measurement can identify true negatives (correctly detect persons with real body temperature lower than the threshold). However, the distribution of the data according to the threshold of 37 °C does not make it possible to evaluate the S-S curves. Thus, an artificial APT threshold of 36.5 °C was established to simulate the S-S curves, which are shown in Figure 14 for the eyes maximum temperature and in Figure 15 for the forehead temperature.

The S-S curves in Figure 14 show a standard trade-off between the sensitivity, which decreases with the increasing IRT threshold, and specificity, which increases with the increasing IRT threshold. The balance between the sensitivity and specificity is important and can be influenced by specific needs. It can be, for example, a request for the maximum success rate in a detection of true positives, which moves the IRT threshold to lower values and increases the number of false positives.

One of the methods of how to find an optimal S-S ratio is the maximum value of the Youden index [20] (i.e., sensitivity plus specificity minus 1). The maximum value of the Youden index is well defined for the maximum eyes temperature in Figure 14. It is obtained for the IRT threshold of 36.4 °C, which leads to sensitivity/specificity of about 0.70/0.69. In contrast, the position of the Youden index maximum is not so clear for the forehead temperature in Figure 15. The maximum can be found at the IRT threshold 36.5 °C and it leads to sensitivity/specificity of about 0.67/0.47. The results are influenced by the small range of the APT and the usage of the artificial APT threshold 36.5 °C. Nevertheless, it can be concluded that the maximum eyes temperature, at given conditions, can provide sensitivity/specificity about 0.7 with a very good indication of the S-S ratio optimum. It is therefore much more suitable for human temperature measurement/screening than the forehead temperature examination, which provides lower S-S values. It is also shown that the optimum IRT threshold can differ from the true body temperature (APT) threshold depending on the optimum selection methodology and requirements.

**Figure 16:**
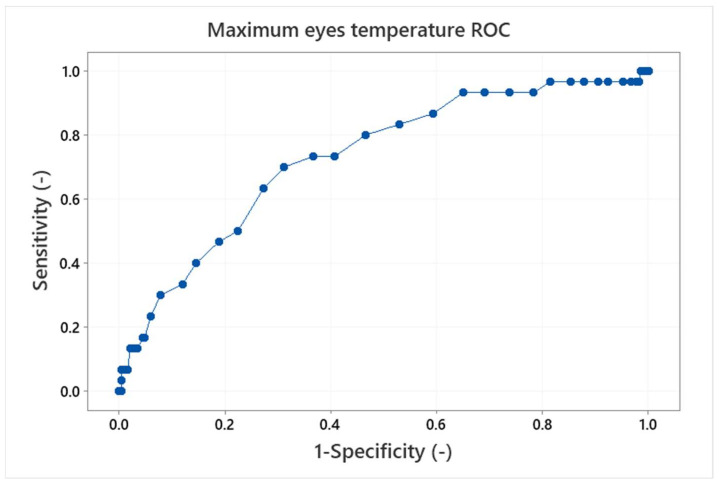
Receiver operation curve (ROC) for the maximum eyes temperature and the APT threshold 36.5 °C. The area under curve is 0.72.

**Figure 17:**
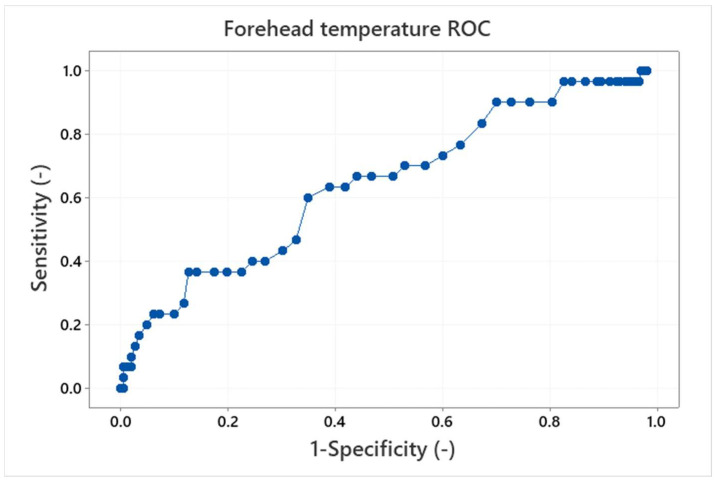
Receiver operation curve (ROC) for the forehead temperature and the APT threshold 36.5 °C. The area under curve is 0.64.

Finally, the receiver operating characteristics (ROC), which are often used for interpretation of diagnostics tests [16,33] can be made. The ROC curve is a plot of true positive versus false positive results. It is a graphical representation of the ability of the IRT threshold (classifier) to discriminate between the true positives and true negatives, i.e., it shows the performance of the classifier. The ROC curves for the maximum eyes temperature and forehead temperature with the APT threshold of 36.5 °C are shown in Figure 16 and Figure 17.

The perfect classifier should hug along the point (0,1) (upper left corner), on the other hand, a diagonal line represents that the classifier is just making random guesses. Comparison of the ROC curves for the eyes maximum and forehead temperature classifiers shows better performance for the eyes maximum temperature examination as the forehead temperature ROC curve tends to be in a the diagonal line. It can be quantified by the area under the curve (AUC) values. The AUC ranges in values from 0 to 1, where a perfect classifier will have an AUC of 1 and a random classifier will have an AUC of 0.5. The AUC for the maximum eyes temperature is 0.72; that means there is a 72% chance that the maximum eyes temperature IRT classifier will be able to distinguish between the positive and negative results (i.e., testers with a body temperature above/below the APT threshold). In contrast, the AUC for the forehead temperature is 0.64 and the curve is not as smooth as in the case of the maximum eyes temperature examination. It is not the worst case (AUC = 0.5), however, it shows that the forehead temperature examination is closer to a random prediction and performs worse than the maximum eyes temperature IRT measurement.

It is necessary to reiterate that the presented results are related to the conditions of the experiment and evaluation procedure. The extended analyses of the sensitivity, specificity and ROC allowed some estimation of the statistical characteristics of the two IRT human temperature measurement methods, their comparison and evaluation of their performance in relation to the APT measurement. The results are however affected by the narrow range of the real body temperatures and by the absence of real true positive testers, which required defining the artificial APT threshold of 36.5 °C. Thus, it is obvious that the performance of the maximum IRT eyes temperature measurement is better than forehead temperature measurement, whose performance is very limited in the assumed measurement range. It is however not possible to conclude that the forehead measurement cannot be used in any case or that the forehead measurement is not capable of identifying a certain number of persons with an elevated body temperature or a fever.

## 6. Conclusions

Statistical analysis of more than 750 measurements by the IR cameras and the armpit thermometer during several months-long periods was carried out. The used measurement configuration did not exactly follow the standard requirements. It often corresponded to practically used configurations of an IR screening, however, the experiment conditions were very stable (e.g., all testers were thermally stabilized).

Despite the quite stable conditions and a blackbody reference calibration, there were differences in the mean values up to 0.5 °C between the APT and IRT eyes maximum temperature measurement and several degrees between the APT and IRT forehead temperature measurement. Differences between individual IR cameras were also observed. This indicated that the IRT camera measurement can hardly be an equivalent to a standard medical body temperature measurement without specific measurement conditions and additional calibrations. Thus, a mean values normalization was suggested and applied in this study to be able to perform additional statistical evaluations.

The mean value and standard deviation of the armpit temperature measurement were 36.16 ± 0.38 °C. It was concluded that, in the case of the IRT measurement, the eyes maximum or face maximum temperature should be evaluated in persons without covering over their eyes (e.g., by glasses). The forehead temperature was however also evaluated in this study as it is a very usual location for human temperature determination by handheld thermometers or IR cameras. The IRT measurement standard deviations observed in this study were 0.4–0.9 °C and 0.7–0.9 °C for the eyes maximum and forehead temperature, respectively (based on the used IR camera). Additionally, the number of outliers was indicated for both IRT and APT measurements.

The extended statistical evaluation of the results was performed in order to find relationships between the APT and IRT measurements and to estimate the sensitivity, specificity and ROC characteristics of the IRT testing. It was found that it was not possible to find accurate relationship between the IRT and APT measurement because of the small temperature range and high data scatter. A strong relationship between the forehead and maximum eyes temperature measurement was observed. The model however did not fit the data well (R2 = 0.37) because of high scatter. Thus, it can be concluded that the forehead temperature measurement cannot be used for an accurate determination of the eyes maximum temperature or the body temperature. It was also confirmed by the evaluation of the TN/FP in relation to the APT. In the case of eyes maximum temperature measurement, 97.0% of the measurements were in the TN group. In contrast, 88.7% and 11.2% of the measurements were in the TN and FP group, respectively, for the forehead temperature measurement.

As only healthy testers participated in the study, the ROC analyses for the IRT measurement were simulated for the APT threshold of 36.5 °C. It was found that the optimal threshold sensitivity/specificity is about 0.70/0.69 and 0.67/0.47 for the eyes maximum and forehead temperature, respectively. The AUC value of the ROC curve was 0.72 for the eyes maximum temperature. That means there is a 72% chance for the IRT to distinguish between the testers above and below the APT body temperature threshold. The AUC for the forehead temperature measurement is 0.64 and the curve is not smooth. These results again confirm a lower performance of the forehead temperature measurement compared to the eyes maximum temperature (IRT) measurement. This conclusion is consistent with results published, e.g., in [34], where forehead and tympanic temperature measurements using infrared sensors were compared.

The results of this study can be affected by the absence of testers with elevated temperatures or a fever. The inclusion of sick people is however not possible in the conditions considered for this study. These conditions should be partially close to a real human IRT measurement in buildings’ interiors. There were however several limitations for performing the tests. Thus, the results should be assumed as limiting, because any additional degree of freedom (e.g., thermally unstabilized persons) would lead to decreasing in accuracy and reliability of the IRT human body temperature measurement if the same or similar devices were used.

## Figures and Tables

**Table 1:** Locations of the maximum face temperature.

Position	Total Count	Percent (%)	Mean (°C)	Standard Dev. (°C)
Eyes	568	74.4	36.06	0.54
Face other	50	6.6	36.38	0.51
Forehead and Temple	21	2.8	36.09	0.38
Mouth	73	9.6	36.25	0.45
Neck	51	6.7	35.87	0.45
